# The Determination of Triacylglycerols and Tocopherols Using UHPLC–CAD/FLD Methods for Assessing the Authenticity of Coffee Beans

**DOI:** 10.3390/foods12234197

**Published:** 2023-11-21

**Authors:** Lama Ismaiel, Benedetta Fanesi, Anastasiya Kuhalskaya, Laura Barp, Sabrina Moret, Deborah Pacetti, Paolo Lucci

**Affiliations:** 1 Department of Agricultural, Food and Environmental Sciences, Università Politecnica delle Marche, 60131 Ancona, Italy; l.ismaiel@pm.univpm.it (L.I.); b.fanesi@univpm.it (B.F.); a.kuchalskaja@univpm.it (A.K.); d.pacetti@univpm.it (D.P.); 2 Department of Agri-Food, Animal and Environmental Science, Università degli Studi di Udine, 33100 Udine, Italy; laura.barp@uniud.it (L.B.); sabrina.moret@uniud.it (S.M.)

**Keywords:** coffee adulteration, charged aerosol detector, triacylglycerols, vitamin E, authenticity

## Abstract

The authenticity of coffee beans was addressed in this study using an analytical method with minimal sample preparation to achieve simple oil extraction and through the implementation of cost-effective equipment. For this purpose, methods using UHPLC with CAD and FLD detectors were applied to detect triglycerides and tocopherols in coffee, respectively. The coffee samples included two main varieties: Arabica from Brazil, Colombia, Ethiopia, and Uganda, as well as the Robusta variety from Cambodia, Guatemala, India, and Vietnam. The samples were either in their green state or subjected to different roasting levels. The used methods successfully distinguished the Arabica and Robusta variants targeted in this study based on their tocopherols and TAG profiles, with the latter being particularly effective for discriminating the origins of the Arabica coffee, while tocopherols excelled at differentiating the origin of the Robusta coffee. TAGs and tocopherols were not affected by the type of roasting, from medium to very dark, suggesting it is possible to distinguish between coffee varieties independently from their degree of roasting. The obtained results hold valuable implications for future research regarding coffee fraud and authenticity.

## 1. Introduction

Consistent consumption of high-quality coffee not only enhances physical performance but also mitigates the risk of various health issues. Coffee serves as a primary affordable dietary source of polyphenols and phenolic acids, which are closely associated with potent antioxidant effects, weight management, boosted vigilance, effectiveness in managing hypertension, and potential anticancer properties. Green coffee beans have been suggested as a candidate to promote a healthy lifestyle and positively influence emotional and mood changes [1]. Females in their middle age who consumed one to two cups of coffee per day had a 17% higher likelihood of achieving the recommended 500 metabolic equivalent (MET) minutes per week according to physical activity guidelines. Consuming caffeine via coffee could potentially help in facing exhaustion and low energy levels, thus possibly overcoming an obstacle that prevents people from engaging in moderate-to-vigorous physical activity (MVPA) [2].

Coffee ranks among the most widely consumed beverages globally, with almost 166,346 thousand 60 kg bags consumed in 2020 [3]. Arabica (*Coffea arabica*) and Robusta (*Coffea canephora*) are the primary coffee bean species used in production. These species differ not only in their appearance but also in various other attributes such as their origin, taste, caffeine levels, and cost [4,5]. Various environmental, agronomic, and additional processing factors exert an influence on the physicochemical attributes of both Robusta and Arabica coffee. In addition, coffee quality is impacted significantly and equally by the pre-harvest practices and post-harvest procedures (40%) followed during the export handling process (20%) [5]. The chemical composition, flavor, and dry matter of coffee are primarily influenced by the level of roasting, which can range from light to medium to dark brown and is visible in the external color of the beans [6]. Robusta green coffee beans show nearly double the antioxidant activity of Arabica green coffee beans. However, this contrast significantly decreases once the beans are roasted [7]. Arabica is generally recognized for its higher coffee quality, and is known for its superior flavor and lower bitterness when compared to Robusta [8,9]. This explains the higher price of the Arabica variety. Coffee is typically sold as a blend of these two types in different proportions, and its price is usually connected to its level of Arabica content. 

Nevertheless, there is a possibility that commercial coffee products may sometimes contain less Arabica than what their labels claim [10]. This raises a challenge, not only for researchers but also for the industry: to detect coffee fraud. To date, the authenticity of coffee has been addressed in plenty of studies [11,12,13,14]. Developing a convenient method for discriminating between Arabica and Robusta is imperative to introduce superior coffee quality traits without adulteration. Liquid chromatography (LC) has been employed in plenty of studies on coffee and its composition. Different detection systems coupled to HPLC have also been used for various analyses. Some examples of such methods are listed as follows.

A comparative analysis of the metabolomic profiles between fermented Arabica and Robusta green coffee beans was conducted using orbitrap LC–MS/MS [15]. Moreover, one study reported comprehensive lipid profiling using LC–MS/MS to determine the origin of valuable Indonesian coffee [16]. Conducting this lipidomic profiling to distinguish between the geographical origins of coffee from various regions in Indonesia led to promising outcomes; the lipids’ characteristics showed potential in discerning the coffee’s origins, suggesting the identification of potential markers [16]. Further, the quantification of caffeine, chlorogenic acid, and trigonelline in cold brew coffee was conducted via high-performance liquid chromatography coupled with a UV detector at 276 nm [17]. Green Arabica and Robusta coffee beans were characterized via their lipid content using liquid chromatography coupled to ion mobility quadrupole time-of-flight mass spectrometry (LC–IM-qTOF-MS) [18]; the lipid profiles of Arabica and Robusta coffee exhibited notable differences, as was evident from the PCA score plots of the data obtained in both positive and negative ionization modes, which clearly indicated a significant separation between the two groups [18]. Tandem mass spectrometry (HPLC–MS/MS) was employed in the quantification of bioactive compounds in coffee silverskin from roasted beans [19]. Ultra-performance liquid chromatography in conjunction with time-of-flight tandem mass spectrometry (UPLC–TOF-MS/MS) allowed for the accurate analyses of triglycerides (TAGs) in green coffee [20]. TAGs are significant constituents of the lipids found in coffee oil and they represent approximately 75% of the total lipids in coffee beans; thus, they were considered chemical descriptors for coffee authentication [13]. In one study, the tocopherol profile of 100% Arabica medium-roasted coffee beans was analyzed using HPLC with a fluorescence detector (FLD) [14]. The differences in tocopherol composition provide convenient means to distinguish between coffee varieties, even after the roasting stage [13]. Using UHPLC–FLD, a fast separation of tocopherols was achieved in less than four minutes [21]. Tocopherols are one of the most important natural antioxidants and enhance the protection of coffee lipids from oxidative damage [12]. Tocopherols, along with tocotrienols, make up the group of eight vitamers that compose vitamin E. Alpha-, beta-, and gamma-tocopherols were detected in both green and roasted Arabica and Robusta coffee samples; the chromatographic profiles of these tocopherols exhibit notable differences, particularly in the case of beta- and gamma-tocopherols [13]. One study determined the caffeine and chlorogenic acid in green and roasted coffee samples using HPLC equipped with a diode array detector (DAD) [22]. Another recently developed approach adopting HPLC coupled with a Corona detector (CAD) enabled the determination of chlorogenic acid, caffeine, caffeic acid, coumaric acid, and ferulic acid [23]. Four rare sugars, namely D-sorbose, D-allose, D-tagatose and D-allulose, in coffee were recovered from coffee samples using HPLC coupled with Corona Veo RS Charged Aerosol Detector (CAD) [24]. UHPLC–CAD resulted in enhanced separation of TAGs, along with significant reductions in both solvent usage and analysis duration, offering exceptional and extensive linearity, sensitivity, and precision. A strong linearity was attained, with correlation coefficients exceeding 0.975 [25].

The innovation of our study lies in the use of HPLC–CAD/FLD for simple, cost-efficient sample preparation and the possible discrimination of green and roasted coffee beans originating from different countries based on their triglyceride and tocopherol contents.

In this study, we investigated the TAGs and tocopherols of coffee by applying an easy method that requires minimal sample preparation and relatively cheap instrumentation, namely UHPLC coupled with CAD and FLD. The advantages of the analytical approaches employed in this study rely on reducing the sample preparation time to one extraction step, without the need for any additional purification procedures. This may not only help to increase the precision of the measurements, but may also improve the sample analysis throughput. TAGs and tocopherols were used to discriminate between Arabica and Robusta species, among different countries of origin, and among different roasting levels, ranging from light to very dark. The coffee used in this study belonged to Brazil, Colombia, Ethiopia, and Uganda, for the Arabica variety. On the other hand, the coffee of the Robusta variety originated from Cambodia, Guatemala, India, and Vietnam.

## 2. Materials and Methods

### 2.1. Chemicals and Reagents

Acetone (ACE), acetonitrile (ACN), isopropanol (IPA), and methanol (MeOH) (all HPLC grade) were purchased from Sigma–Aldrich (Milano, Italia). Triglyceride standards, namely 1,2,3-Trilinoleoylglycerol (LLL); 1,2,3-Trioleoylglycerol (OOO); 1,3-Dioleoyl-2-palmitoylglycerol (OPO); 1,3-Dipalmitoyl-2-oleoylglycerol (POP); and 1,2-Dioleoyl-3-palmitoyl-rac-glycerol (OOP), and Tocopherols (α, β, and δ) were purchased from Sigma–Aldrich (Milano, Italia).

### 2.2. Coffee Samples

For the analysis, we selected 8 samples with various geographical origins (Table S1). Out of these, 4 samples were *Coffea arabica* samples from Brazil, Colombia, Ethiopia, and Uganda. The remaining 4 samples were *Coffea canephora* var. Robusta from Cambodia, Guatemala, India, and Vietnam. All samples were supplied by a local industrial coffee roaster (Caffè del Faro Robin s.r.l., Montegranaro, Italy) who were able to confirm their botanical and geographical origins, as well as their classification as green (G), light (L), medium (M), dark (D), and very dark (D++) bean samples based on their degree of roasting.

### 2.3. Oil Extraction

The oil extraction of the coffee samples was carried out using a Soxhlet extractor. An aliquot of 50 g of coffee samples was finely ground and extracted with *n*-hexane for 5 h. The extract was then dried over anhydrous sodium sulphate and *n*-hexane was evaporated by means of a vacuum rotary evaporator for further UHPLC analysis.

### 2.4. Analysis of Triglycerides

Triglycerides were determined using UHPLC–CAD following the parameters reported by Lucci et al. [25], with some modifications to the mobile phase gradients and column temperature. Chromatographic separation was carried out using an Ascentis Express C18 column (150 × 2.1 mm, 2.7 μm particle size). Gradient elution was achieved from solvent A (ACE:IPA, 70:30, *v*/*v*) and solvent B (ACN) with a flow of 0.5 μL min^−1^, as follows: 0–10 min, isocratic condition at 45% A; 10–20 min, linear gradient from 45 to 50% A; 20–25 min, linear gradient from 50 to 60% A; 25 min, back to initial conditions at 45% A; and from 25 to 30 min, isocratic step at 45% A. The column temperature was 30 °C. The injected volume for each sample was 1 μL. CAD parameters were as follows: power function: 1.65; data collection rate: 10 Hz; filter: 3.6; evaporation temperature: 50 °C. Equivalent carbon numbers (ECNs) were used to define the elution order of the TAGs [26,27]. The Thermo Scientific™ Dionex™ Chromeleon™ 7.2 SR4, Milan, Italy was used for data acquisition and analysis. Results were expressed as the total area of each TAG and as a percentage of the internal areas. TAGs were tentatively identified based on the analytical standards available and by comparing the chromatogram with those of well-known oil samples (i.e., olive oil, sunflower oil, corn oil). For evaluating the linearity of the UHPLC–CAD methodology, LLL was injected at different levels ranging from 50 to 5000 ng on the column. As result, a good linearity was found within the tested range (y = 2.1196x − 0.1301), with a correlation coefficient higher than 0.9995. The uniformity of response factor for different TAGs was also tested by injecting on the column the same amount (1 µL) of TAG standards reported in Section 2.1, including LLL, and comparing the peaks area achieved. As has already been reported [25], a satisfactory uniformity of response factor was obtained with CAD, with relative differences in terms of TAG areas lower than 6% among the different TAG response factors when compared to the LLL reference area.

### 2.5. Analysis of Tocopherols

Tocopherol analysis was carried out using a validated UHPLC method already reported by Lucci et al. [21]. A fluorescence RF-20Axs detector was employed, with excitation and emission wavelengths set at 296 nm and 325 nm, respectively, at a frequency of 10 Hz. The samples were diluted in IPA to a concentration of 100 mg mL^−1^, and 1 μL was injected. Separation was accomplished using an Agilent Eclipse PAH column (1.7 μm particle size, 2.1 mm × 50 mm) with isocratic elution, employing a mobile phase composed of MeOH–ACE (60:40, *v*/*v*) with a flow rate of 600 μL min^−1^ throughout the run. The oven’ temperature was maintained at 30 °C. Tocopherols (α, β, and δ) were quantified using three distinct calibration curves, as described by Lucci et al. [21], and the results were expressed as mg/kg of coffee oil.

### 2.6. Statistical Analysis

Experimental results of the performed analysis were processed by the MetaboAnalyst 5.0 online platform. A normalized and scaled dataset was used for total visualization. Principle component analysis (PCA), heatmaps, and variable importance in projection (VIP) were employed to monitor the variations in the TAG and tocopherols profiles of Arabica and Robusta coffee samples with diverse origins and roasting levels. Statistically significant differences between Arabica and Robusta were shown using an ANOVA test.

## 3. Results

**Figure 1:**
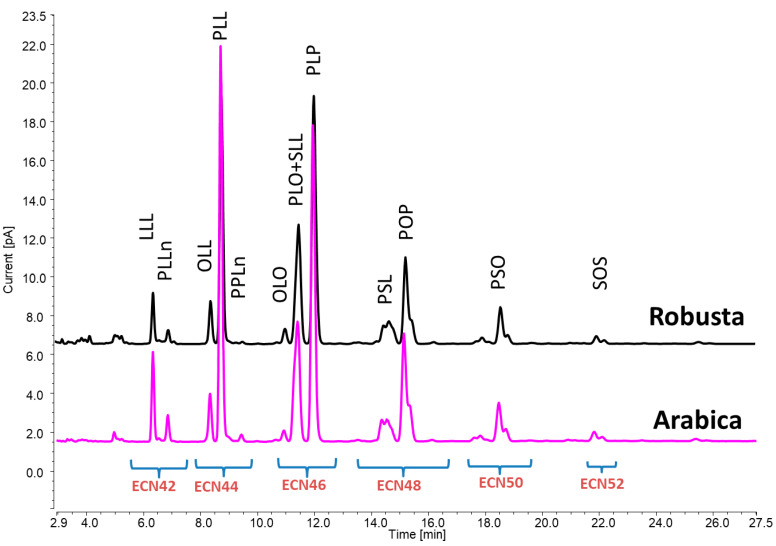
UHPLC–CAD chromatograms showing the TAG profiles of Arabica coffee (in pink) and Robusta coffee (in black). TAGs are annotated with the initials of the common names for their fatty acids; the list of acronyms used for FAs is as follows: P = (C16:0) palmitic acid; Po = (C16:1) palmitoleic acid; S = (C18:0) stearic acid; O = (C18:1) oleic acid; L = (C18:2) linoleic acid; Ln = (C18:3) linolenic acid; A = (C20:0) arachidic acid; ECN = equivalent carbon number.

Tocopherols and TAGs were employed as chemical descriptors for a straightforward method to primarily distinguish between a variety of Robusta and Arabica coffee. Secondly, the analytical potential of the proposed methodologies was also tested for discriminating between Arabica and Robusta coffee belonging to various origins and/or subjected to different roasting levels. From an analytical point of view, the advantages of the analytical approach employed in this study rely on its minimal sample manipulation, which was reduced to one extraction step without the need for any additional purification procedure. This may not only help to increase the precision of the measurements by reducing the number of sample preparation steps but also may improve the sample analysis throughput. In fact, once the oil is extracted from the matrix, the samples can be directly injected into UHPLC–CAD/FLD systems. At the same time, it should be pointed out that the methodologies herein proposed were intentionally based on less expensive detectors compared to other equipment configurations (i.e., LC coupled with mass spectrometry) and those that do not require skilled personnel. These aspects are of particular relevance when developing methods intended to be used for the authentication of food samples. As a result, a four-minute UHPLC–FLD method enabled the distinct identification of α-, β-, and δ-tocopherols, while for the TAG analysis, the use of gradient elution with UHPLC–CAD via a column packed with smaller particles allowed us to obtain a clear separation of TAGs, especially those in the ECN42 and ECN44 groups, which usually co-elute when using alternative detectors, such as a refractive index, that prevent changes in mobile phase compositions during analysis. Twelve TAGs were tentatively identified in the following order: LLL, PLLn, OLL, PLL, PPLn, OLO, PLO + SLL, PLP, PSL, POP, and SOS (Figure 1). The dataset of tocopherols and TAGs (Table S2) was subsequently used for further discrimination analysis.

**Figure 2:**
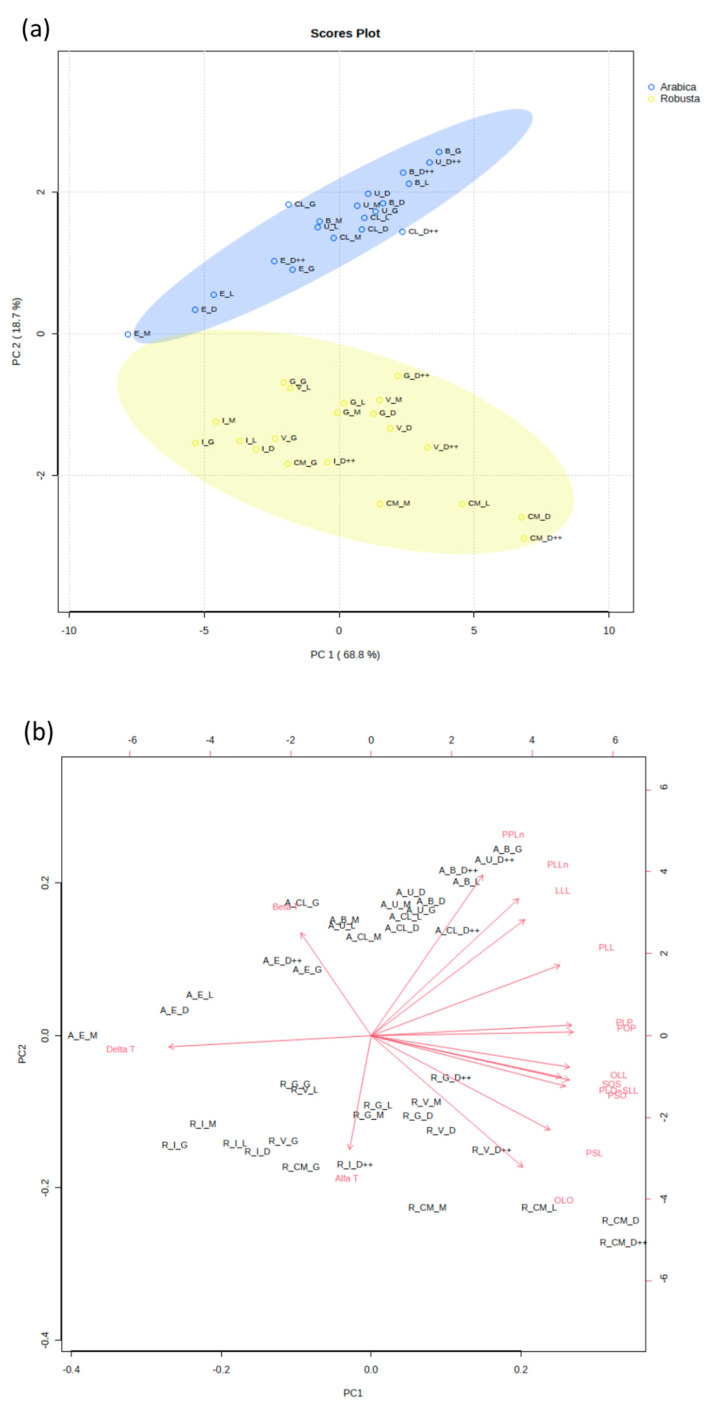
Observed differences in the TAG and tocopherol profiles of Arabica and Robusta coffee samples. (**a**) Principal component analysis (PCA) of the TAG and tocopherol content in Arabica coffee (blue) and Robusta coffee (yellow) samples. Each dot represents a distinct sample type. (**b**) Loading plots from the principal component analysis. Letters G, L, M, D, and D++ depict the samples’ roasting level, namely green, light, medium, dark, and very dark, respectively. Letters CM, G, V, I, E, CL, U, and B depict the origins of the samples, namely Cambodia, Guatemala, Vietnam, India, Ethiopia, Colombia, Uganda, and Brazil, respectively. Letters A and R represent the types of coffee, namely Arabica and Robusta.

To differentiate between the two varieties of coffee, we conducted an analysis of their triacylglycerol and tocopherol profiles. Based on the triacylglycerol and tocopherol level, Arabica coffee variants form clearly separate groups from Robusta coffee variants, regardless of their country of origin or their level of roasting. PC1 and PC2 explain 87.5% of the total observed variation across variables (Figure 2a). We noticed significantly elevated levels of PPLn, β-tocopherol, PLLn, and LLL in the Arabica coffee samples, whereas the levels of OLO and PSL were notably higher in the Robusta coffee samples (Figure S1). Differences in the levels of PPLn and β-tocopherol drive the main observed variation among Arabica and Robusta samples. PC1 correlated positively with the profiles of all the identified TAGs, while the level of α-, β-, and δ-tocopherols exhibited a negative correlation (Figure 2b).

**Figure 3:**
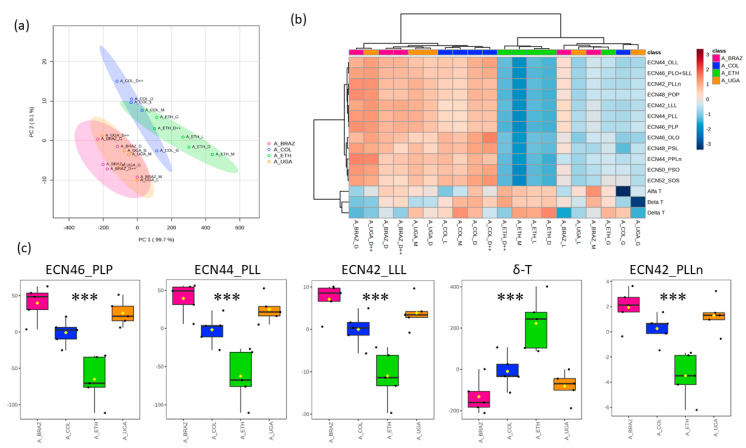
Observed differences across Arabica coffee samples in their TAG and tocopherol profiles. (**a**) Principal component analysis (PCA) of TAG and tocopherol content in Arabica coffee from Brazil (pink), Arabica coffee from Colombia (blue), Arabica coffee from Ethiopia (green), and Arabica coffee from Uganda (orange). (**b**) Heatmap representing the TAG and tocopherol profiles across Arabica coffee samples with different origins. (**c**) Average TAG and tocopherol values in the samples of Arabica coffee of different origins. Significances are indicated as * < 0.05, ** < 0.01, and *** < 0.001, calculated using the ANOVA test.

Further, we investigated the profiles of Arabica coffee samples with different origins, such as Brazil, Colombia, Ethiopia, and Uganda. PCA1 and PCA2 explained 99.8% of the total observed variation across these samples (Figure 3a). The TAG levels exhibited a broad range, leading to a substantial coefficient of variation ranging from 36.6% to 44.4%, while the coefficient of variation in the tocopherol levels spanned from 12.7% to 30.7% (Table S2). In the supplementary table, TAGs are reported as the collective area’s mean values; while tocopherols are reported as the mean of the concentration (mg/kg) of coffee oil. The samples were grouped according to their level of identified TAGs and tocopherol content. Interestingly, the samples from Ethiopia constituted a unique cluster, characterized by lower TAG levels and higher tocopherol levels when compared to the coffee samples with other origins (Figure 3b). We observed notable increases in the levels of all identified TAGs, with a particularly pronounced rise in ECN46_PLP, ECN44_PLL, ECN42_LLL, and ECN42_PLLn in the coffee samples from Brazil, Colombia, and Uganda. In contrast, the coffee samples from Ethiopia exhibited a significantly higher level of δ-tocopherol (Figure 3c). In addition, the level of other TAGs was significantly lower in the coffee samples originating from Ethiopia (Figure S2). The latter shows variations in the TAG and tocopherol profiles of Arabica and Robusta coffee samples with diverse origins and roasting levels. The relative concentrations of the corresponding TAG and tocopherol in each coffee sample are also presented in Figure S2. Moreover, the weighted sum of absolute regression coefficients, represented by the variable importance in projection (VIP), is reported as well.

**Figure 4:**
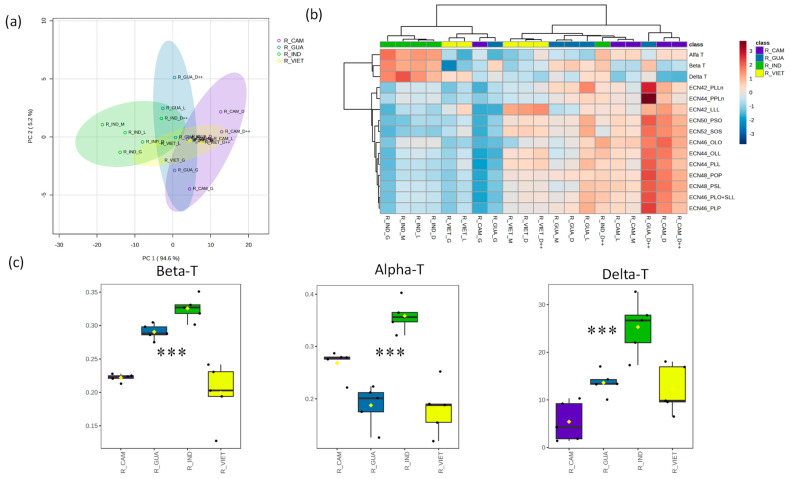
Observed differences across Robusta coffee samples in their TAG and tocopherol profiles. (**a**) Principal component analysis (PCA) of TAG and tocopherol content in Robusta coffee from Cambodia (purple), Guatemala (blue), India (green), and Vietnam (yellow). (**b**) Heatmap representing the TAG and tocopherol profiles across Robusta coffee samples with different origins. (**c**) Average TAG and tocopherol values in the samples of Robusta coffee of different origins. Significances are indicated as * < 0.05, ** < 0.01, and *** < 0.001, calculated using the ANOVA test.

Next, we analyzed the composition of Robusta coffee samples originating from Cambodia, Guatemala, India, and Vietnam. A combination of PCA1 and PCA2 accounted for 99.8% of the total observed variability among these samples (Figure 4a). Their TAG levels spanned a wide range, resulting in a substantial coefficient of variation fluctuating from 28.5% to 61.1%, while the coefficient of variation for their tocopherol levels ranged from 22.1% to 63.4% (Table S2). These samples were clustered based on their respective levels of identified TAGs and tocopherol content. Notably, the samples from India formed a distinct cluster, characterized by lower TAG levels and higher tocopherol levels compared to the coffee samples from the other regions (Figure 4b). We observed remarkable and statistically significant increases in the levels of all identified tocopherols in the coffee samples from India. Conversely, the coffee samples originating from Vietnam displayed significantly lower levels of α- and β-tocopherols (Figure 4c).

**Figure 5:**
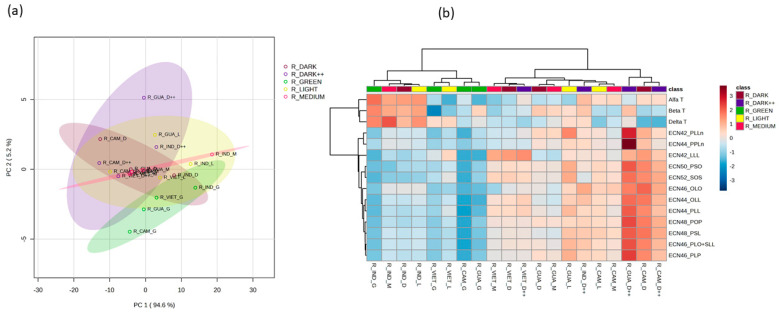
Observed variations in TAG and tocopherol profiles across the Robusta coffee samples with different roasting levels. (**a**) Principal component analysis (PCA) of TAG and tocopherol content of different roasting degrees of Robusta coffee. (**b**) Heatmap representing the TAG and tocopherol profiles across Robusta coffee samples with different degrees of roasting: green (green), light (yellow), medium (pink), dark (violet), and very dark (purple).

We also investigated how the degree of roasting in coffee beans affects their composition of TAGs and tocopherols. In our analysis of Arabica coffee samples, we found that the roasting level had no significant impact on the TAG or tocopherol levels (Figure S3). In contrast, the roasting level of the Robusta coffee beans influenced their TAG and tocopherol profiles. PC1 and PC2 explain 99.8% of the total observed variation (Figure 5a). Samples with no or light roasting (green or light) tended to cluster together based on their respective levels of identified TAGs and tocopherols, while samples with a higher degree of roasting formed a distinct cluster (Figure 5b). The coffee samples that were not subjected to any roasting exhibited significantly lower levels of all TAGs (Figure S4). The latter shows the observed variation in the TAG and tocopherol profiles across the Robusta coffee samples with different roasting levels.

## 4. Discussion

A total separation of the Arabica and Robusta coffee samples was achieved and strongly classified into two split groups using PCA (Figure 2). The latter expressed 87.5% variations via PC1 and PC2, with a clear separation of both varieties’ profiles in terms of their TAGs and tocopherols. Independently from the roasting level, Arabica samples of Ethiopian origin has the largest ellipse of the graphs, while Cambodian coffee comprises the main ellipse of the Robusta samples. Comparable results were observed for both Arabica and Robusta coffee by González et al. [13]; they computed the PCA of the TAG profile, which accounted for up to 56.1% of the total variance between Arabica and Robusta coffee from diverse origins [13]. The composition of TAGs and tocopherols in Arabica and Robusta coffee samples can be linked to the origin of these samples, depending on their specific soil and climate characteristics [14]. Of the top variables identified, ECN44_PPLn, ECN42_PLLn, β-tocopherol, ECN42_LLL, ECN46_OLO, and ECN48_PSL can be considered significant contributors to the differences in the TAG and tocopherol profiles of the studied Arabica and Robusta coffee. TAG profile and β- and γ-tocopherols were effectively used to differentiate between coffee varieties by González et al. [13]. The mean percentages of ECN42_LLL and ECN46_OLO were helpful in distinguishing between the Arabica and Robusta variants as Arabica exhibited significantly higher and lower contents of these two TAGs, respectively [13]. Our results match these findings, where the ECN42_LLL and ECN46_OLO concentrations were significantly different between Arabica and Robusta. Consequently, this highlights the potential of these TAGs for use in differentiating between Arabica and Robusta.

The type of soil in all four regions of origin for the Arabica coffee is mainly volcanic. Brazil and Uganda possess soils consisting of volcanic loam, as well as having the same altitude (1000–1100 m) [14,28]. This resemblance explains the similar cluster of TAGs and tocopherols for the Arabica coffee from Brazil and Uganda in the PCA plot (Figure 3a). Coffee samples originating from Ethiopia and Colombia share higher altitudes (1950–1750 m), respectively. Ethiopia’s mean annual rainfall is 850 mm, much lower than Colombia’s, which is 2562 mm [14]. Therefore, we demonstrate the observed differences across Arabica coffee samples in their TAG and tocopherol profiles, represented with a PCA score plot and heatmap in Figure 3. Using UHPLC–CAD allowed us to achieve a clear separation of TAGs, especially for ECN 42. Consequently, four TAGs, namely ECN42_PLLn, ECN42_LLL, ECN44_PLL, and ECN46_PLP, were found with significant differences (*p* < 0.001) between Arabica coffee samples of various origins. This method was used successfully for olive oil triacylglycerols analysis by Lucci et al. [25]. Ethiopia had significantly lower concentrations of these TAGs. Likewise, similar concentrations of PLLn, LLL, PLL, and PLP were reported by González et al. in Arabica coffee of Ethiopian origin [13]. On the contrary, Arabica coffee originating from Brazil presented with a rich TAG profile, followed by Uganda. Cornelio-Santiago et al. [29] reported a comparable estimation of the TAG composition present in green coffee oil from Brazil. Nevertheless, the analysis of TAG profiles for the Robusta coffee from different origins, i.e., Cambodia, Guatemala, India, and Vietnam, did not exhibit any significant differences. Cossignani et al. [11] were able to effectively utilize TAG analysis data as a valid method for distinguishing 100% authentic Arabica coffee from a blend containing 90% Arabica and 10% Robusta. Thus, the TAG profile is again a useful tool to tentatively distinguish between Arabica and Robusta coffee. In general, coffee lipids, encompassing free fatty acids and triacylglycerols, have been recognized as distinctive markers for distinguishing between Arabica and Robusta coffee [18]. Furthermore, lipid profiling was reported as a potential marker to elucidate the geographical origin of coffee from several regions [16].

Our analysis revealed significantly higher δ-tocopherol levels in the Ethiopian samples, namely of the Arabica coffee, when compared to those from other regions. In the same way, Arabica coffee from Ethiopia recorded high levels of δ-T, β-T, γ-T, and α-T in the study reported by Simedru et al. [14]. The tocopherol profiles of the Robusta coffee expressed significant differences (*p* < 0.001) between the samples from India, which had abundant concentrations, and those from Cambodia, Guatemala, and Vietnam, which had lower ones (Figure 4). These results show that the tocopherol profile could be useful for the discrimination of not only Robusta coffee from different origins but also for the discrimination between Arabica and Robusta variants collected from these regions; however, a larger number of samples is needed to confirm these results. It is important to note that the tocopherol profile has been confirmed to be a potential and valuable tool for distinguishing between Arabica and Robusta coffees, whether in their green or roasted form [12].

The variations in TAG and tocopherol profiles between the Arabica and Robusta coffee of different origins in our study can be fundamentally explained by the diversity of the cultivation conditions in each region, and by the varying post-harvest treatment of the coffee beans [5,30].

No significant effect of roasting on the Arabica coffee samples was noted in either the TAG or tocopherol profiles, regardless of their origin. Similarly, most of the tocopherol remained after the roasting process for Arabica coffee from Hawaii, Brazil, Costa Rica, Jamaica, Colombia, Ethiopia, Honduras, Guatemala, and Kenya [12]. Given the high content of lipid and lipophilic tocopherol in Arabica beans, roasting had no or a minimal impact on them [12,31]. Conversely, the roasting process was found to significantly affect the level of TAGs in the Robusta samples, especially in the cases of certain TAGs, namely ECN44_OLL, ECN46_PLO+SLL, ECN44_PLL, ECN46_OLO, ECN48_POP, ECN48_PSL, ECN46_PLP, ECN42_LLL, and ECN50_PSO (Figure S4). These TAGs exhibited better results after roasting compared to those of the green coffee beans, confirming the results reported by González et al. [13]. A potential explanation for this could be related to the lower amount of antioxidants in Robusta coffee, which allows for a stronger effect on the formation of its TAGs. Interestingly, we were able to distinguish between the Arabica and Robusta coffee targeted in this study regardless of the origin or degree of roasting.

## 5. Conclusions

The analytical method applied in this study aimed to reduce the necessary sample preparation to one simple oil extraction and to use common and cheap instrumentation, such as HPLC–CAD and HPLC–FLD, to detect TAGs and tocopherols, respectively. A four-minute UHPLC–FLD method enabled the distinct identification of α-, β-, and δ-tocopherols, while the use of UHPLC coupled to CAD allowed us to perform a chromatographic separation via gradient elution, obtaining a satisfactory separation of the TAGs. Regardless of the geographical origin of samples, the methodologies proposed herein allowed us to clearly separate Arabica and Robusta coffee depending on their TAG and tocopherol profiles. At the same time, their TAGs and tocopherols were not affected by the type of roasting (from medium to very dark), thus reinforcing the robustness of TAGs and tocopherols as possible coffee identifiers and highlighting the usefulness of the proposed analytical approach for addressing issues concerning the authenticity of variants of coffee. Additionally, TAG profiles have been proven to be particularly effective for discriminating the origins of Arabica coffee, while tocopherols better elucidated the origins of Robusta coffee. Specifically on this last point, however, further studies including a larger and exhaustive sample size are needed for validating the use of these UHPLC–CAD/FLD methods in geographical origin assessments. In conclusion, the results obtained here are handy for future studies regarding the fraud and authenticity of coffee and other food.

## Data Availability

Data supporting the findings of this study will be available on request.

## Supplementary Materials

The following supporting information can be downloaded at: https://www.mdpi.com/article/10.3390/foods12234197/s1.
