# Supplementary material for: The Determination of Triacylglycerols and Tocopherols Using UHPLC–CAD/FLD Methods for Assessing the Authenticity of Coffee Beans
Source: Foods. 2023 Nov 21;12(23):4197. doi: 10.3390/foods12234197 (PMC10706131; doi:10.3390/foods12234197)
Supplement: Supplementary file 1 [file foods-12-04197-s001.zip › foods-2701253-supplementary.pdf]

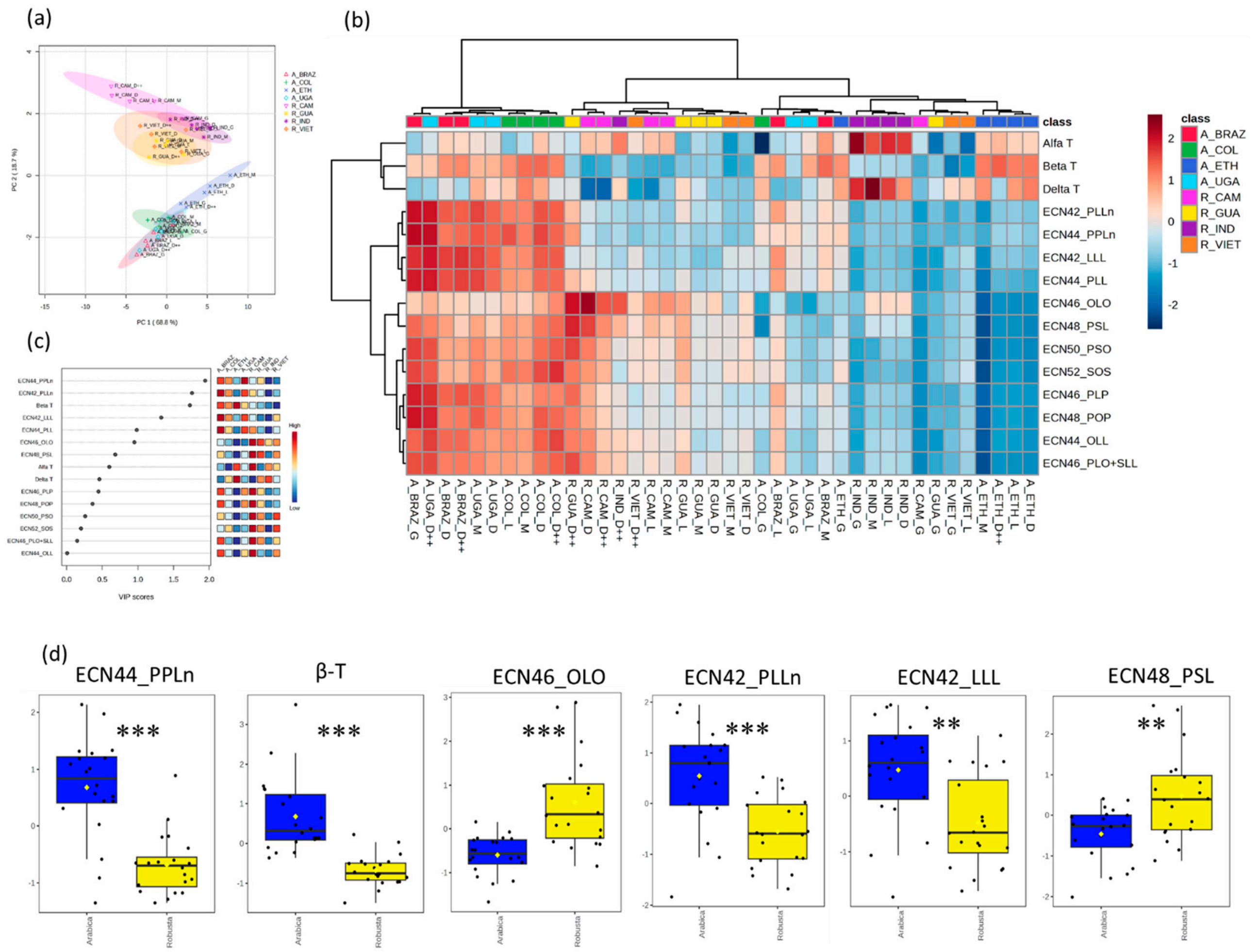

Figure S1. Variation in TAGs and tocopherols profile in arabica and robusta coffee samples with diverse origins and roast levels. (a) Principal component analysis (PCA) of TAG and tocopherol content for different coffee samples representing Arabica coffee from Brazil (pink), Arabica coffee from Colombia (green), Arabica coffee from Ethiopia (blue), and Arabica coffee from Uganda (turquoise), Robusta coffee from Cambodia (violet), Robusta coffee from India (purple), Robusta coffee from Vietnam (orange), Robusta coffee from Guatemala (yellow). (b) Heatmap represents the profile of TAGs and tocopherols across Arabica and Robusta coffee samples with different origin and roasting degree. (c) Plot represented the results of statistical analysis for multi-group data (ANOVA). The colored boxes on the right indicate the relative concentrations of the corresponding TAG and tocopherol in each coffee sample. Variable importance in projection (VIP) is the weighted sum of absolute regression coefficients. (d) Average value of TAGs and tocopherols in the samples of Arabica coffee (blue) and Robusta coffee (yellow). Significances are indicated by * < 0.05, ** < 0.01, *** < 0.001 using ANOVA

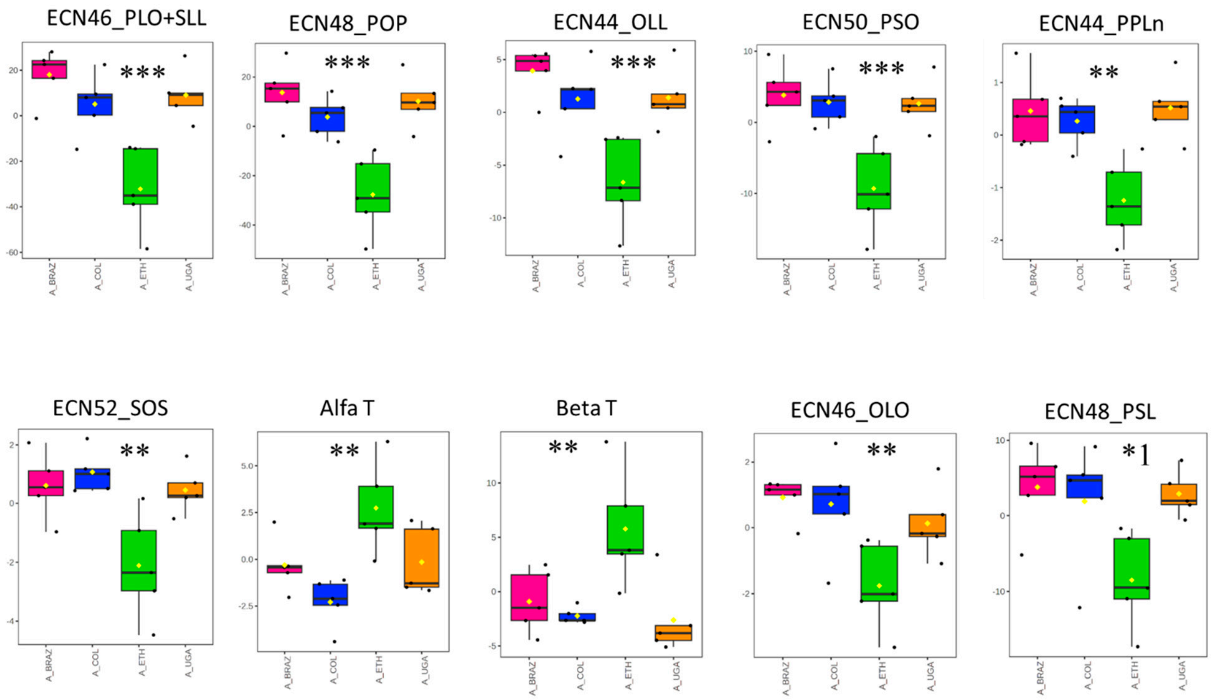

Figure S2. Observed differences across Arabica coffee samples in the profile of TAGs and tocopherols. Average value of TAGs and tocopherols in the samples of Arabica coffee of different origin. Significances are indicated by * < 0.05, ** < 0.01, *** < 0.001 using ANOVA.

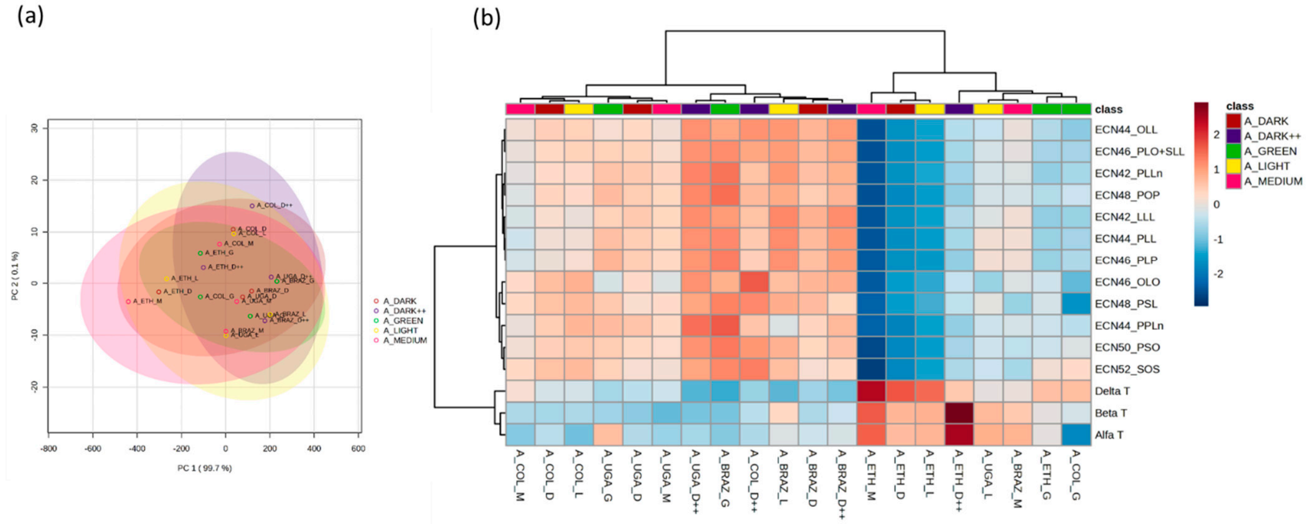

Figure S3. Observed variation in the profile of TAGs and tocopherols across the samples with different roasting degree of Arabica coffee. (a) Principal component analysis (PCA) of TAG and tocopherol content in Arabica coffee with green (green), light (yellow), medium (pink), dark (violet), and very dark (purple) roasting degrees. (b) Heatmap represents the profile of TAGs and tocopherols across Arabica coffee samples with different roasting degrees.

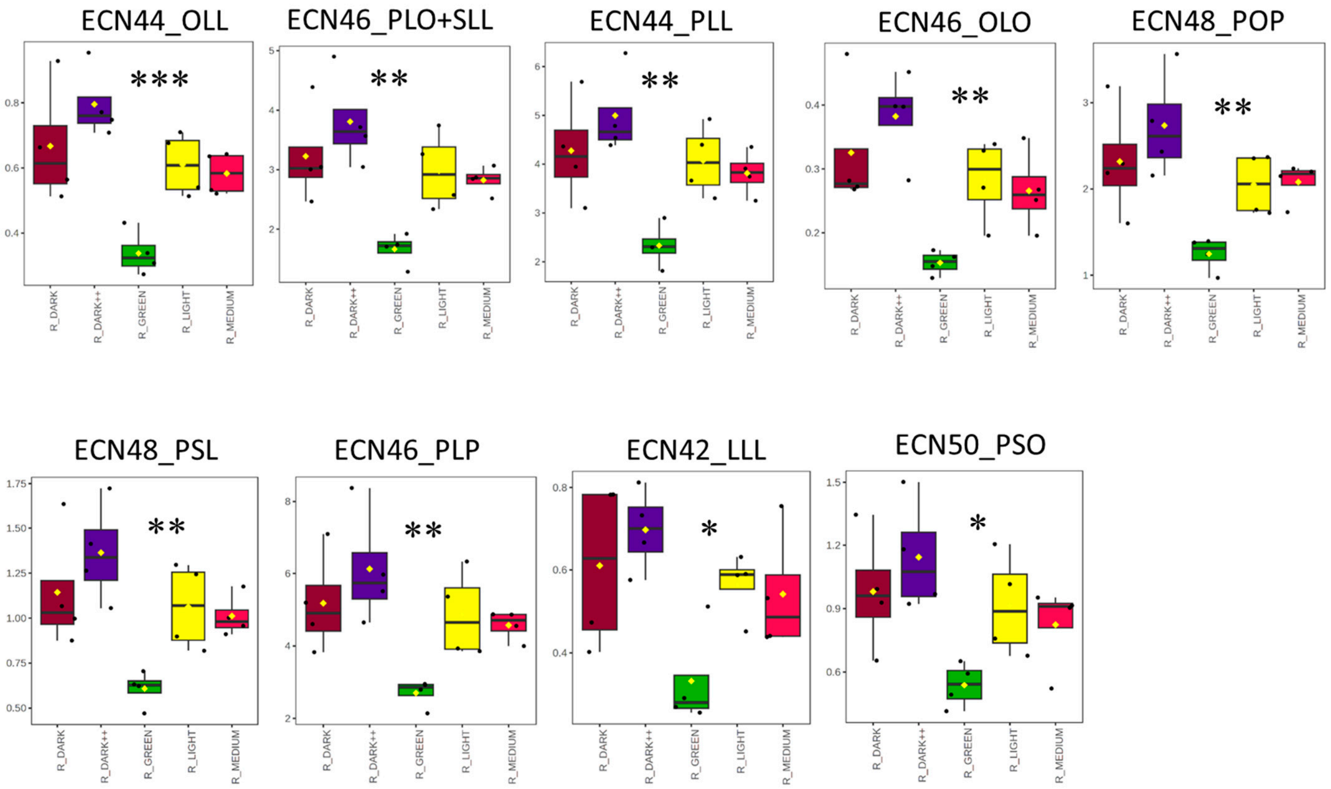

Figure S4. Observed variation in the profile of TAGs and tocopherols across the samples with different roasting degrees of Robusta coffee. Average value of TAGs and tocopherols in the samples of Robusta coffee with different roasting degree. Significances are indicated by * < 0.05, ** < 0.01, *** < 0.001 using ANOVA.

Table S 1. List of coffee samples

Variety	Type	Origin
Coffea arabica	Arabica	Brazil
Coffea arabica	Arabica	Colombia
Coffea arabica	Arabica	Ethiopia
Coffea arabica	Arabica	Uganda
Coffea canephora	Robusta	Cambodia
Coffea canephora	Robusta	Guatemala
Coffea canephora	Robusta	India
Coffea canephora	Robusta	Vietnam

Table S2

Datasets used for statistical analysis; TAGs are reported as collected areas mean values; Tocopherols reported as the mean of the concentration (mg/kg) of coffee oil

	Arabica+Robusta														
	ECN42_LLL	ECN42_PLLn	ECN44_OLL	ECN44_PLL	ECN44_PPLn	ECN46_OLO	ECN46_PLO+SLL	ECN46_PLP	ECN48_PSL	ECN48_POP	ECN50_PSO	ECN52_SOS	Beta T	Alfa T	Delta T
R_CAM_G	0.26	0.07	0.27	1.81	0.02	0.13	1.29	2.14	0.47	0.97	0.41	0.11	0.23	0.22	9.22
R_CAM_L	0.59	0.18	0.68	4.40	0.05	0.34	3.26	5.36	1.24	2.35	1.02	0.24	0.22	0.28	4.27
R_CAM_M	0.53	0.16	0.64	3.91	0.04	0.35	3.07	4.87	1.18	2.24	0.95	0.23	0.22	0.29	10.31
R_CAM_D	0.78	0.23	0.93	5.69	0.06	0.48	4.39	7.09	1.64	3.19	1.35	0.31	0.21	0.28	1.82
R_CAM_D++	0.67	0.20	0.77	4.78	0.05	0.40	3.72	5.97	1.41	2.79	1.18	0.29	0.23	0.28	1.36
R_GUA_G	0.27	0.13	0.31	2.32	0.03	0.15	1.71	2.93	0.63	1.40	0.59	0.15	0.29	0.13	13.30
R_GUA_L	0.59	0.27	0.71	4.92	0.06	0.33	3.74	6.33	1.29	2.37	1.20	0.29	0.28	0.18	17.01
R_GUA_M	0.44	0.20	0.53	3.75	0.04	0.25	2.84	4.87	0.96	2.20	0.90	0.21	0.29	0.21	13.38
R_GUA_D	0.47	0.20	0.56	3.95	0.05	0.28	3.05	5.20	1.00	2.29	0.93	0.21	0.30	0.20	10.05
R_GUA_D++	0.73	0.34	0.95	6.27	0.14	0.45	4.90	8.37	1.72	3.57	1.50	0.33	0.31	0.22	14.27
R_VIET_G	0.51	0.09	0.43	2.89	0.02	0.17	1.92	2.95	0.71	1.38	0.65	0.17	0.13	0.19	16.94
R_VIET_L	0.63	0.13	0.51	3.66	0.03	0.20	2.34	3.85	0.82	1.76	0.76	0.21	0.19	0.12	18.06
R_VIET_M	0.76	0.15	0.64	4.35	0.03	0.20	2.87	4.55	1.00	2.15	0.92	0.24	0.20	0.16	9.83
R_VIET_D	0.78	0.15	0.66	4.36	0.04	0.27	3.01	4.60	1.07	2.18	0.99	0.25	0.23	0.19	9.58
R_VIET_D++	0.81	0.17	0.71	4.54	0.04	0.28	3.05	4.65	1.06	2.15	0.97	0.24	0.24	0.25	6.52
R_IND_G	0.29	0.10	0.34	2.30	0.03	0.16	1.74	2.80	0.62	1.24	0.49	0.12	0.35	0.40	27.78
R_IND_L	0.45	0.15	0.54	3.30	0.04	0.27	2.57	3.93	0.90	1.72	0.68	0.15	0.33	0.37	26.67
R_IND_M	0.44	0.15	0.52	3.25	0.04	0.27	2.52	3.99	0.91	1.73	0.52	0.14	0.33	0.35	32.69
R_IND_D	0.40	0.15	0.51	3.10	0.04	0.27	2.47	3.83	0.88	1.60	0.65	0.14	0.32	0.36	21.99
R_IND_D++	0.58	0.19	0.75	4.39	0.05	0.40	3.57	5.51	1.26	2.43	0.92	0.20	0.30	0.32	17.31
A_ETH_G	0.80	0.25	0.58	4.64	0.10	0.18	2.53	4.57	0.86	2.21	0.84	0.24	0.59	0.25	20.84
A_ETH_L	0.48	0.16	0.32	2.64	0.05	0.10	1.41	2.52	0.45	1.18	0.43	0.12	0.61	0.27	21.64
A_ETH_M	0.18	0.06	0.12	1.03	0.02	0.04	0.54	0.99	0.16	0.43	0.15	0.04	0.61	0.28	22.07
A_ETH_D	0.43	0.14	0.29	2.43	0.04	0.09	1.32	2.41	0.41	1.03	0.37	0.10	0.62	0.27	23.39
A_ETH_D++	0.50	0.15	0.33	2.76	0.05	0.10	1.46	2.57	0.46	1.14	0.43	0.11	0.65	0.29	11.58
A_COL_G	0.76	0.25	0.48	4.42	0.09	0.12	2.34	4.51	0.41	2.18	0.83	0.23	0.51	0.07	19.46
A_COL_L	1.26	0.42	0.92	7.21	0.17	0.30	4.17	7.33	1.36	3.37	1.28	0.34	0.59	0.19	18.47
A_COL_M	1.28	0.42	0.90	7.07	0.15	0.28	4.04	7.10	1.32	3.26	1.24	0.33	0.63	0.22	23.04

A_COL_D	1.47	0.47	1.02	8.03	0.17	0.32	4.55	7.94	1.47	3.85	1.39	0.36		0.64	0.28	20.58
A_COL_D++	1.46	0.46	1.07	8.06	0.16	0.36	4.73	7.93	1.52	3.74	1.45	0.38		0.61	0.24	14.61
A_UGA_G	0.83	0.27	0.50	4.97	0.10	0.13	2.48	5.14	0.77	2.21	0.75	0.18		0.33	0.22	9.07
A_UGA_L	0.76	0.23	0.46	4.57	0.08	0.12	2.19	4.58	0.68	1.82	0.64	0.16		0.55	0.25	12.60
A_UGA_M	1.54	0.49	0.93	8.73	0.17	0.26	4.39	8.65	1.34	3.83	1.32	0.32		0.52	0.25	20.09
A_UGA_D	1.44	0.46	0.89	8.07	0.16	0.26	4.15	8.04	1.18	3.57	1.22	0.29		0.53	0.24	16.83
A_UGA_D++	1.69	0.54	1.08	9.47	0.20	0.32	4.93	9.39	1.44	4.26	1.47	0.35		0.49	0.23	11.13
A_BRAZ_G	1.60	0.55	1.01	9.39	0.20	0.29	4.89	9.68	1.51	4.38	1.51	0.37		0.48	0.20	9.80
A_BRAZ_L	1.27	0.38	0.79	7.08	0.09	0.21	3.58	7.03	1.04	2.90	1.01	0.24		0.58	0.21	8.41
A_BRAZ_M	0.98	0.29	0.63	5.65	0.10	0.18	2.82	5.55	0.66	2.25	0.75	0.18		0.64	0.31	15.53
A_BRAZ_D	1.55	0.47	0.99	8.49	0.15	0.29	4.47	8.44	1.34	3.56	1.21	0.29		0.58	0.27	14.99
A_BRAZ_D++	1.60	0.46	1.00	8.77	0.16	0.28	4.48	8.73	1.16	3.59	1.23	0.29		0.60	0.26	11.72
median	0.74	0.20	0.64	4.48	0.05	0.27	3.03	5.01	1.02	2.23	0.93	0.24		0.34	0.25	14.80
st deva	0.44	0.14	0.26	2.28	0.06	0.10	1.17	2.24	0.39	0.99	0.36	0.09		0.17	0.07	6.94
coefficient of variation	0.60	0.72	0.40	0.51	1.10	0.38	0.39	0.45	0.38	0.45	0.39	0.36		0.50	0.27	0.47
%	59.73	72.32	40.11	50.99	110.09	38.30	38.78	44.68	37.82	44.64	38.52	36.45	0.00	49.56	26.66	46.86

	Robusta														
	ECN42_LLL	ECN42_PLLn	ECN44_OLL	ECN44_PLL	ECN44_PPLn	ECN46_OLO	ECN46_PLO+SLL	ECN46_PLP	ECN48_PSL	ECN48_POP	ECN50_PSO	ECN52_SOS	Beta T	Alfa T	Delta T
R_CAM_G	0.26	0.07	0.27	1.81	0.02	0.13	1.29	2.14	0.47	0.97	0.41	0.11	0.23	0.22	9.22
R_CAM_L	0.59	0.18	0.68	4.40	0.05	0.34	3.26	5.36	1.24	2.35	1.02	0.24	0.22	0.28	4.27
R_CAM_M	0.53	0.16	0.64	3.91	0.04	0.35	3.07	4.87	1.18	2.24	0.95	0.23	0.22	0.29	10.31
R_CAM_D	0.78	0.23	0.93	5.69	0.06	0.48	4.39	7.09	1.64	3.19	1.35	0.31	0.21	0.28	1.82
R_CAM_D++	0.67	0.20	0.77	4.78	0.05	0.40	3.72	5.97	1.41	2.79	1.18	0.29	0.23	0.28	1.36
R_GUA_G	0.27	0.13	0.31	2.32	0.03	0.15	1.71	2.93	0.63	1.40	0.59	0.15	0.29	0.13	13.30
R_GUA_L	0.59	0.27	0.71	4.92	0.06	0.33	3.74	6.33	1.29	2.37	1.20	0.29	0.28	0.18	17.01
R_GUA_M	0.44	0.20	0.53	3.75	0.04	0.25	2.84	4.87	0.96	2.20	0.90	0.21	0.29	0.21	13.38
R_GUA_D	0.47	0.20	0.56	3.95	0.05	0.28	3.05	5.20	1.00	2.29	0.93	0.21	0.30	0.20	10.05
R_GUA_D++	0.73	0.34	0.95	6.27	0.14	0.45	4.90	8.37	1.72	3.57	1.50	0.33	0.31	0.22	14.27
R_VIET_G	0.51	0.09	0.43	2.89	0.02	0.17	1.92	2.95	0.71	1.38	0.65	0.17	0.13	0.19	16.94

R_VIET_L	0.63	0.13	0.51	3.66	0.03	0.20	2.34	3.85	0.82	1.76	0.76	0.21	0.19	0.12	18.06
R_VIET_M	0.76	0.15	0.64	4.35	0.03	0.20	2.87	4.55	1.00	2.15	0.92	0.24	0.20	0.16	9.83
R_VIET_D	0.78	0.15	0.66	4.36	0.04	0.27	3.01	4.60	1.07	2.18	0.99	0.25	0.23	0.19	9.58
R_VIET_D++	0.81	0.17	0.71	4.54	0.04	0.28	3.05	4.65	1.06	2.15	0.97	0.24	0.24	0.25	6.52
R_IND_G	0.29	0.10	0.34	2.30	0.03	0.16	1.74	2.80	0.62	1.24	0.49	0.12	0.35	0.40	27.78
R_IND_L	0.45	0.15	0.54	3.30	0.04	0.27	2.57	3.93	0.90	1.72	0.68	0.15	0.33	0.37	26.67
R_IND_M	0.44	0.15	0.52	3.25	0.04	0.27	2.52	3.99	0.91	1.73	0.52	0.14	0.33	0.35	32.69
R_IND_D	0.40	0.15	0.51	3.10	0.04	0.27	2.47	3.83	0.88	1.60	0.65	0.14	0.32	0.36	21.99
R_IND_D++	0.58	0.19	0.75	4.39	0.05	0.40	3.57	5.51	1.26	2.43	0.92	0.20	0.30	0.32	17.31
median	0.55	0.16	0.60	3.93	0.04	0.27	2.94	4.62	1.00	2.17	0.92	0.21	0.26	0.24	13.34
st dev	0.17	0.06	0.18	1.12	0.02	0.10	0.90	1.52	0.33	0.64	0.29	0.06	0.06	0.08	8.45
coefficient of variation	0.31	0.39	0.31	0.29	0.61	0.37	0.31	0.33	0.33	0.30	0.31	0.30	0.22	0.34	0.63
%	31.32	39.29	30.57	28.55	61.12	36.72	30.59	32.86	32.77	29.57	31.44	29.96	22.05	34.22	63.36

ARABICA															
A_ETH_G	0.80	0.25	0.58	4.64	0.10	0.18	2.53	4.57	0.86	2.21	0.84	0.24	0.59	0.25	20.84
A_ETH_L	0.48	0.16	0.32	2.64	0.05	0.10	1.41	2.52	0.45	1.18	0.43	0.12	0.61	0.27	21.64
A_ETH_M	0.18	0.06	0.12	1.03	0.02	0.04	0.54	0.99	0.16	0.43	0.15	0.04	0.61	0.28	22.07
A_ETH_D	0.43	0.14	0.29	2.43	0.04	0.09	1.32	2.41	0.41	1.03	0.37	0.10	0.62	0.27	23.39
A_ETH_D++	0.50	0.15	0.33	2.76	0.05	0.10	1.46	2.57	0.46	1.14	0.43	0.11	0.65	0.29	11.58
A_COL_G	0.76	0.25	0.48	4.42	0.09	0.12	2.34	4.51	0.41	2.18	0.83	0.23	0.51	0.07	19.46
A_COL_L	1.26	0.42	0.92	7.21	0.17	0.30	4.17	7.33	1.36	3.37	1.28	0.34	0.59	0.19	18.47
A_COL_M	1.28	0.42	0.90	7.07	0.15	0.28	4.04	7.10	1.32	3.26	1.24	0.33	0.63	0.22	23.04
A_COL_D	1.47	0.47	1.02	8.03	0.17	0.32	4.55	7.94	1.47	3.85	1.39	0.36	0.64	0.28	20.58
A_COL_D++	1.46	0.46	1.07	8.06	0.16	0.36	4.73	7.93	1.52	3.74	1.45	0.38	0.61	0.24	14.61
A_UGA_G	0.83	0.27	0.50	4.97	0.10	0.13	2.48	5.14	0.77	2.21	0.75	0.18	0.33	0.22	9.07
A_UGA_L	0.76	0.23	0.46	4.57	0.08	0.12	2.19	4.58	0.68	1.82	0.64	0.16	0.55	0.25	12.60
A_UGA_M	1.54	0.49	0.93	8.73	0.17	0.26	4.39	8.65	1.34	3.83	1.32	0.32	0.52	0.25	20.09
A_UGA_D	1.44	0.46	0.89	8.07	0.16	0.26	4.15	8.04	1.18	3.57	1.22	0.29	0.53	0.24	16.83
A_UGA_D++	1.69	0.54	1.08	9.47	0.20	0.32	4.93	9.39	1.44	4.26	1.47	0.35	0.49	0.23	11.13
A_BRAZ_G	1.60	0.55	1.01	9.39	0.20	0.29	4.89	9.68	1.51	4.38	1.51	0.37	0.48	0.20	9.80

A_BRAZ_L	1.27	0.38	0.79	7.08	0.09	0.21	3.58	7.03	1.04	2.90	1.01	0.24	0.58	0.21	8.41
A_BRAZ_M	0.98	0.29	0.63	5.65	0.10	0.18	2.82	5.55	0.66	2.25	0.75	0.18	0.64	0.31	15.53
A_BRAZ_D	1.55	0.47	0.99	8.49	0.15	0.29	4.47	8.44	1.34	3.56	1.21	0.29	0.58	0.27	14.99
A_BRAZ_D++	1.60	0.46	1.00	8.77	0.16	0.28	4.48	8.73	1.16	3.59	1.23	0.29	0.60	0.26	11.72
median	1.26	0.40	0.84	7.07	0.13	0.23	3.81	7.07	1.10	3.08	1.11	0.27	0.59	0.25	16.18
st deviation	0.47	0.15	0.31	2.59	0.06	0.10	1.39	2.61	0.44	1.18	0.42	0.10	0.07	0.05	4.98
coefficient of variation	0.37	0.37	0.36	0.37	0.44	0.41	0.37	0.37	0.40	0.38	0.37	0.39	0.13	0.20	0.31
%	37.03	37.16	36.40	36.63	44.41	40.73	36.62	36.98	40.42	38.28	37.34	38.6<br>8	12.7<br>4	19.7<br>7	30.78
